# HbSC disease: Not as benign as you think

**DOI:** 10.1002/hem3.70311

**Published:** 2026-03-31

**Authors:** Edeghonghon Olayemi

**Affiliations:** ^1^ Richmond Hospital, Vancouver Coastal Health Richmond British Columbia Canada

Hemoglobin C (HbC) arises from a missense mutation in the beta Hb (HBB) gene, leading to the replacement of glutamic acid with lysine in the beta globin chain. This mutation is very common in West Africa, where it is believed to have originated, providing some protection against malaria.^1^, ^2^ HbC is less soluble than normal HbA, causing intra‐cellular HbC crystal formation, which increases blood viscosity and shortens red cell lifespan. HbS is another hemoglobin mutant that causes sickle cell disease (SCD) when both alleles are mutated (HbSS), which is the most common form of SCD. Globally, compound heterozygosity for HbS and HbC (HbSC) is the second most common SCD genotype.

In both forms of SCD, the lifespan of red blood cells is significantly decreased compared to healthy individuals (average 120 days) and is about 30 days in HbSC and 20 days in HbSS. Compared to anemia and hemolysis, hyperviscosity plays a disproportionate role in HbSC morbidity. In Ghana, HbSC makes up 30%–40% of people living with SCD, which is comparable to 30% in the United States and the United Kingdom. Globally, it is estimated that between 55,000 and 100,000 babies are born with HbSC annually.^3^, ^4^

Due to the delayed onset of complications, people living with HbSC disease tend to have a longer life expectancy than those living with HbSS disease. This is largely influenced by demographics, with those living in high‐income countries surviving longer than those in low‐ and middle‐income countries (LMICS), where the majority of people with the disease reside. Thus, while HbSC disease has an overall milder clinical phenotype compared to HbSS disease, this is not always the case. Unfortunately, due to this wrong assumption, people living with HbSC disease were until recently usually excluded from clinical trials for disease‐modifying therapies. For example, hydroxyurea (HU) was approved by the US Food and Drug Administration (FDA) for the management of adults living with HbSS disease in 1998. For decades afterwards, there were no specific disease‐modifying therapies for those living with HbSC disease, and physicians were often not sure if therapies like HU approved for HbSS could be extended to HbSC. Similarly, in 2023, the US FDA approved gene therapy for the management of SCD patients 12 years and older with recurrent vaso‐occlusive crises. The clinical trials for the approved agents were primarily conducted in people with HbSS disease and HbS‐β° thalassemia, again, excluding those living with HbSC disease.

A retrospective study from Ghana found that approximately 10% of individuals with HbSC had one or more severe complications that if they had HbSS would have qualified them for HU therapy by the American Society for Hematology (ASH) criteria.^5^ In that study, pain and acute chest syndrome (ACS) incidence rates among individuals with HbSC were 74.6 (95% CI, 67.9–81.3) and 2.3 (95% CI, 1.2–3.5) events per 100 patient‐years, respectively. It is obvious that like the more common and better‐studied HbSS, people living with HbSC disease also suffer complications such as vaso‐occlusion, chronic organ damage, and early death. However, they continue to receive less specialized care compared to those with HbSS disease.

Certain SCD complications are more common in HbSC than in HbSS. These include proliferative sickle retinopathy (PSR), which is the most common complication of HbSC disease. In a study by Downes et al, by the age of 26 years, PSR had occurred in 43% of individuals with HbSC disease compared to 14% with HbSS disease.^6^ Priapism is seen in about 20% of men with HbSC disease, often occurring as they reach sexual maturity,^7^ and may be the presenting clinical feature of HbSC in previously undiagnosed adolescents and young adults with HbSC disease. Others include osteonecrosis, ACS and pulmonary hypertension. On the other hand, complications like stroke, which are less frequent in HbSC, tend to peak in older adults, in whom hemorrhagic stroke is more common. Women with HbSC disease, like all women with SCD, are at increased risk of maternal and fetal morbidity and mortality. While newborn screening has been shown to reduce SCD morbidity and mortality through early preventive care, health care workers are often at a loss as to what to offer newborns with HbSC disease, for example, there is no clear direction with regards to use of penicillin prophylaxis in HbSC, which is known to be beneficial in HbSS. Most children with HbSC disease may live without any specialized management until they develop complications such as priapism, stroke, or proliferative retinopathy, some of which are irreversible.

For too long, HbSC disease was erroneously thought of as essentially benign, leading to a poorer level of care. Health care workers did not know how best to manage people living with the disease. However, with the increased availability of information about HbSC, there is no reason for this level of care to continue, because we know that people living with HbSC disease may present with some of the same complications seen in those with HbSS disease. It is heartening to note that clinical trials on novel therapies in SCD now recruit HbSC participants. Clinical trial investigators, the pharmaceutical industry, and institutional review boards (IRBs) owe people living with HbSC disease a duty to ensure they are not left out of any breakthrough therapy for SCD.

Finally, in addition to ongoing attempts at establishing newborn screening programmes in LMICs, it may be helpful to at the same time implement health campaigns that encourage people to voluntarily know their Hb phenotype/genotype status. Systems could be designed to offer counseling and screening for SCD at specific points where people make contact with health care facilities such as during immunizations, antenatal care, and family planning clinics. This will help identify those who were not diagnosed during the newborn period. Similarly, health care workers should have a high index of suspicion when they encounter patients with otherwise unexpected complications frequently seen in SCD, such as priapism, stroke, retinopathy, and proteinuria, and they should be ready to offer necessary assistance towards diagnosis and referral to specialists as applicable.

## AUTHOR CONTRIBUTIONS

**Edeghonghon Olayemi**: Conceptualization; writing—original draft; writing—review and editing.

## CONFLICT OF INTEREST STATEMENT

The author declares no conflicts of interest.

## FUNDING

This research received no funding.

## Data Availability

Data sharing is not applicable to this article as no datasets were generated or analyzed during the current study.
